# Neurocognitive predictors of progression from late-life depression to all-cause dementia and Alzheimer’s disease: a systematic review and meta-analysis

**DOI:** 10.1192/j.eurpsy.2025.736

**Published:** 2025-08-26

**Authors:** M. Bocharova, R. Desai, A. John, G. Charlesworth, J. Stott, A. Young, D. Aarsland

**Affiliations:** 1 Centre for Healthy Brain Ageing, Institute of Psychiatry, Psychology and Neuroscience; 2 ADAPT lab, Department of Clinical, Educational and Health Psychology, University College London; 3 Centre for Affective Disorders, Institute of Psychiatry, Psychology and Neuroscience, London, United Kingdom

## Abstract

**Introduction:**

Late-life depression (LLD) is consistently linked with higher risk of subsequent dementia (Livingston G et al. Lancet 2024; 404: 572-628); however, predictors of progression from LLD to dementia are yet to be identified.

**Objectives:**

In this systematic review and meta-analysis, we focus specifically on cognitive phenotypes of LLD as predictors of progression to dementia. This review aims to summarize findings from longitudinal studies exploring which cognitive domains affected by LLD are more likely to predict progression to all-cause dementia and Alzheimer’s Disease.

**Methods:**

MEDLINE, Embase and PsycINFO were searched for relevant studies published by April 18^th^, 2024. Study search and selection, data extraction and risk of bias assessment were performed by two reviewers separately, in accordance with the updated Preferred Reporting Items for Systematic Reviews and Meta-Analysis (PRISMA) 2020 guidelines (Page MJ et al. BMJ 2021; 372). Effect sizes for performance on neurocognitive tests were extracted and pooled separately for all-cause dementia and Alzheimer’s disease (AD) outcomes.

**Results:**

Six studies were selected for inclusion (See Fig. 1 for PRISMA flowchart). Conversion from LLD to all-cause dementia was strongly predicted by worse performance on delayed recall (SMD 0.84 [0.64 – 1.05]), immediate recall (SMD 1.02 [0.63 – 1.41]), attention/working memory (SMD 1.17[0.82 – 1.52]), processing speed (SMD 1.23 [0.37 – 2.10]), delayed recognition (SMD 1.30 [0.59 – 2.01]) and orientation (SMD 1.13 [0.90 – 1.36]); and moderately by verbal fluency (SMD 0.70 [0.50 – 0.91]), naming (SMD 0.54 [0.16 – 0.93]), and construction (SMD 0.67 [0.37 – 0.98]), but not intelligence (see Fig. 2). For AD, strong effects were observed for deficits in delayed recall (SMD 1.30 [0.59 – 2.01]), immediate recall (SMD 1.26 [0.74-1.79]), and orientation (SMD 1.64 [0.67 – 2.62]), and small to moderate for verbal fluency (SMD 0.47 [0.06 – 0.87]) and processing speed (0.66 [0.26 – 1.07]); attention or naming were not significant predictors (See Fig. 3).

**Image 1:**

**Figure:**
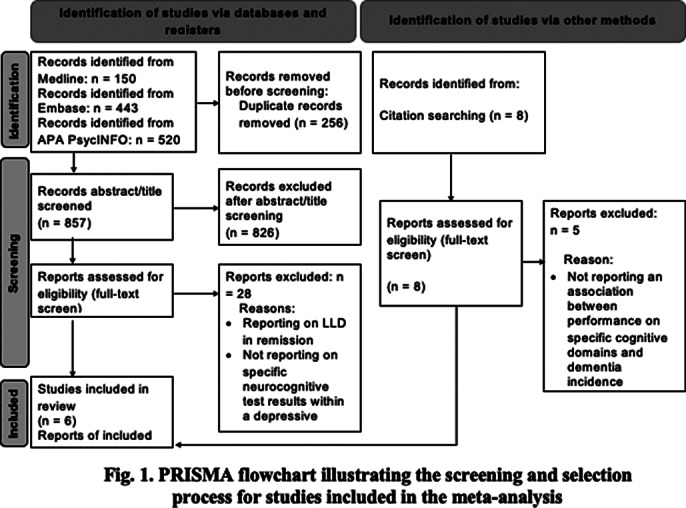

**Image 2:**

**Figure:**
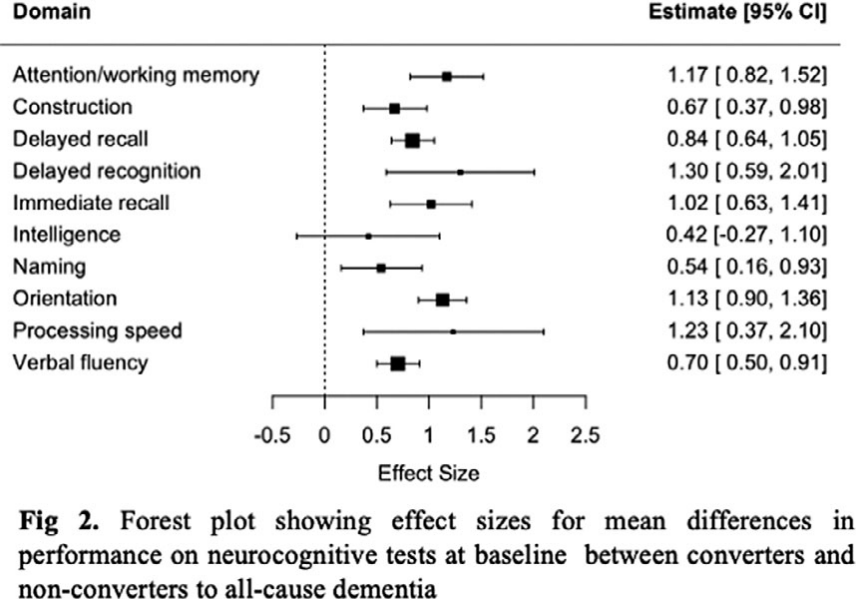

**Image 3:**

**Figure:**
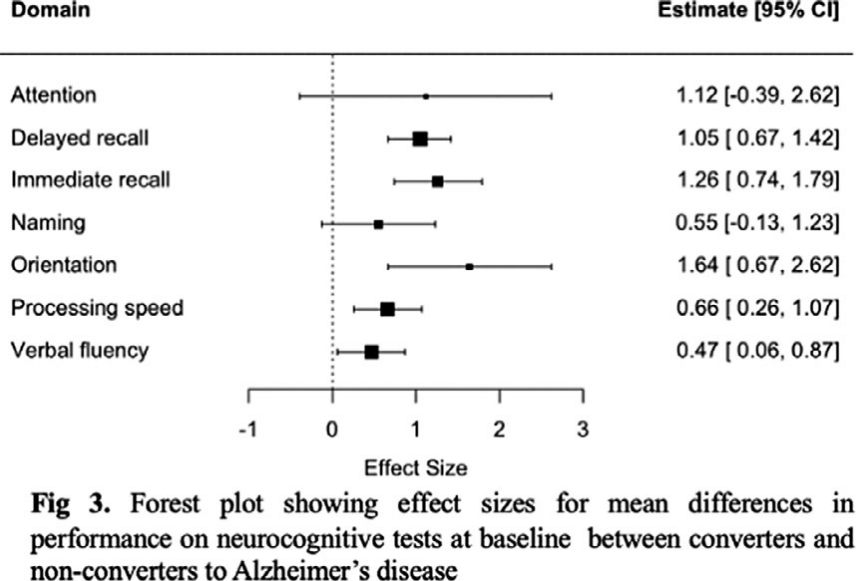

**Conclusions:**

There appear to be significant neurocognitive differences across a wide range of domains within an episode of LLD between those who convert to dementia and those who do not. Future studies should aim to establish a neurocognitive profile of LLD associated with risk of conversion to specific subtypes of dementia.

**Disclosure of Interest:**

None Declared

